# LFMNet: a lightweight model for identifying leaf diseases of maize with high similarity

**DOI:** 10.3389/fpls.2024.1368697

**Published:** 2024-04-23

**Authors:** Jian Hu, Xinhua Jiang, Julin Gao, Xiaofang Yu

**Affiliations:** ^1^ College of Computer and Information Engineering, Inner Mongolia Agricultural University, Hohhot, China; ^2^ Key Laboratory of Agricultural and Animal Husbandry Big Data Research and Application, Inner Mongolia Autonomous Region, Hohhot, China; ^3^ Department of Information Engineering, Inner Mongolia Technical College of Mechanics and Electrics, Hohhot, China; ^4^ College of Agricultural, Inner Mongolia Agricultural University, Hohhot, China

**Keywords:** identification of maize leaf diseases, lightweight model, multi-level, complex background, attention mechanism

## Abstract

Maize leaf diseases significantly impact yield and quality. However, recognizing these diseases from images taken in natural environments is challenging due to complex backgrounds and high similarity of disease spots between classes.This study proposes a lightweight multi-level attention fusion network (LFMNet) which can identify maize leaf diseases with high similarity in natural environment. The main components of LFMNet are PMFFM and MAttion blocks, with three key improvements relative to existing essential blocks. First, it improves the adaptability to the change of maize leaf disease scale through the dense connection of partial convolution with different expansion rates and reduces the parameters at the same time. The second improvement is that it replaces a adaptable pooling kernel according to the size of the input feature map on the original PPA, and the convolution layer to reshape to enhance the feature extraction of maize leaves under complex background. The third improvement is that it replaces different pooling kernels to obtain features of different scales based on GMDC and generate feature weighting matrix to enhance important regional features. Experimental results show that the accuracy of the LFMNet model on the test dataset reaches 94.12%, which is better than the existing heavyweight networks, such as ResNet50 and Inception v3, and lightweight networks such as DenseNet 121,MobileNet(V3-large) and ShuffleNet V2. The number of parameters is only 0.88m, which is better than the current mainstream lightweight network. It is also effective to identify the disease types with similar disease spots in leaves.

## 1 Introduction

Maize is not only one of the most important food crops in China, but also a vital raw material for animal husbandry and light industry. However, diseases are the main factors that affect corn production, and the annual production loss is 6–10% (Zeng et al., 2022b). In order to reduce the loss and improve the yield and quality of maize, it is essential and necessary to use advanced technology to monitor and provide early warning of maize diseases (Sunil et al., 2020). It is reported that there are more than 80 kinds of maize diseases in the world and more than 30 kinds in China. Currently, the common and serious diseases are rust, curvularia leaf spot, gray leaf spot, northern leaf blight, brown spot, and southern leaf blight (Zhang et al., 2018). These diseases affect the growth and development of maize and reduce the disease resistance and yield. The identification and classification of maize diseases is the basis and key of maize disease monitoring and early warning. However, the identification and classification of maize diseases face many difficulties and challenges. On the one hand, the location of maize disease is scattered, a variety of lesions coexist, the lesion area is small, and there are diseases with similar spot characteristics, which easily cause large recognition error. On the other hand, the image of maize leaf disease collected under natural conditions has a complex background environment and causes interference, which poses some difficulties to the visual and accurate identification of maize disease.

The traditional maize leaf disease identification method relies on agricultural technicians to perform detection on site, this method is not only time-consuming, but also has a high equipment cost, and the results are not real, so it is unable to carry out disease control (Wu, 2021) in time. In order to help farmers identify maize leaf diseases quickly, effectively and accurately, we need a convenient and fast application algorithm, which is of great significance to improve maize yield.

The continuous change of machine vision technology provides a new idea for the detection of maize leaf diseases. In recent years, deep learning technology has been widely used in agricultural disease recognition, especially convolution neural network (CNN), which is a powerful and efficient method, that provides a strong driving force for the classification and recognition of maize disease images. Bhatt et al. (2019) used adaptive enhancement algorithm and decision tree-based strategy to improve the classifier in a variety of CNN architectures (VGG 16,Inception v3,ResNet 50), which can identify three kinds of maize leaf diseases with high similarity. Jiang et al. (2021) proposed a multitasking classification method for rice diseases based on VGG16, which overcomes the problem of over-fitting and minimizes the loss. The classification accuracy of rice dataset and wheat dataset is 97.22% and 98.75%, respectively. Chen et al. (2020a) used transfer learning to improve the final output layer of VGG network for plant disease detection. They feed the feature information extracted from the VGG network to the Inception module to obtain the final classification probability. The results show that the average classification accuracy of the model on the mixed dataset of corn and rice is 92.00%. Wu (2021) used the pre-training model of resnet50 and vgg19 to preserve the trained convolution layer to build a dual-channel model. They splice to construct a full connection layer, flatten the feature map, and achieve a recognition accuracy of 98.33% for three kinds of maize diseases. Liu and Zhang (2022) proposed an Inception-V3-based transfer learning method to address the small sample size problem of the training data. The results of the method with pathological images show a promising performance with an accuracy of 99.45 ± 0.17%. In addition, Zeng et al. (2022a) improved ResNet50 by replacing convolution kernel, activation function and loss function, and propose SKPSNet-50 model, which classifies a corn disease dataset in real environment with an accuracy of 92.6%. Shamsul rayhan chy et al. (2023) integrated convolutional neural networks (CNN), DenseNet201 and an improved CNN model with random depth through ensemble learning. The new model combine three different networks to achieve the best performance. The average accuracy of the model in the maize leaf disease image of Plant Village dataset is 98.36%. Liu et al. (2021) proposed a method to identify plant diseases that highlights some of the characteristics of the disease. The designed module divides the image, calculates the weight of each block, calculates the weighted loss function using the weight, classifies the features using LSTM network, and achieves 99.78% recognition accuracy on the PlantVillage dataset. Arumuga arun and Umamaheswari (2023) proposed a multi-crop disease detection method using point-by-point and standard convolution block cascade, and reach a detection accuracy of 98.14%. However, these deep learning methods have a large number of network model parameters, and the network model design is complex, which limits their application ability in mobile devices.

With advances in the Internet ofThings, mobile platforms such as moblie inspection robots make precision agriculture develop quickly (Ye et al., 2023; Tang et al., 2024). Due to the conflict between the high computational power requirements of the models and the limited computational power of plant protection equipment, it is a challenging task to deploy efficient and lightweight plant disease detection models on mobile platforms. Chen et al. (2020b) proposed a new lightweight network model called Mobile-DANet to identify maize diseases. The recognition accuracy of the model is 98.5% on the open corn dataset with simple background and 95.86% on the local corn disease dataset with complex background. Chen Y. et al. (2022) proposed a lightweight corn disease recognition model called DFCANet, which relies on dual-feature fusion and downsampling module fusion of deep and shallow features, suppressing background noise and focusing on the lesion area. The recognition efficiency of 5 kinds of maize leaf diseases reaches 96.63%. Zeng et al. (2022b) proposed a lightweight dense scale network, which uses expansion convolution to improve the adaptability to the change of maize leaf disease scale. The number of parameters only accounts for 45.4% of the minimum number of parameters in the comparison model (ShuffleNet V2 ~ 1.3m), and the accuracy on the test dataset is 95.4%. Anita Shrotriya et al. (2023) proposed a lightweight neural network model, which uses depth separable convolution and expansive convolution to extract focus disease features while reducing the number of parameters, and finally achieves a high accuracy of 97.73% on PlantVillage datasets. Lin et al. (2022) proposed a lightweight CNN model called GrapeNet for identifying different symptom stages of specific grape diseases. Compared with DenseNet 121, which has the highest accuracy in the classical network model, the number of parameters of GrapeNet is reduced by 4.81 million. As a result, the training time of GrapeNet is about two times less than that of DenseNet 121.The accuracy on the test dataset is 86.29%. These lightweight neural network models can show good performance in plant leaf disease image recognition under complex background. However, under the complex background, the characteristics of many kinds of maize leaf diseases are highly similar, such as northern leaf spot, curvularia leaf spot and southern leaf blight, so the actual classification effect of these models is greatly affected.

Attention mechanism is an effective method to extract detail features. There have been many studies on attention mechanism with impressive results (Hu et al., 2018; Woo et al., 2018; Zhao et al., 2020; Hou et al., 2021; Zhang et al., 2022). Among them, Hu et al. (2018) proposed a novel architectural unit, that adaptively recalibrates channel-wise feature responses by explicitly modelling inter dependencies between channels, called Squeeze-and-Excitation(SE). Woo et al. (2018) proposed a simple effective attention module for feed-forward convolutional neural networks called CBAM. SE and CBAM can be seamlessly integrated into any CNN architecture and applied to image recognition. With the lightweight design of the model, the attention mechanism is improved towards simplicity and efficiency. Hou et al. (2021) proposed a novel attention mechanism for mobile networks by embedding positional information into channel attention called coordinate attention(CA). This model is simple and can be flexibly plugged into classic mobile networks that reduced parameters and improved accuracy. Li et al. (2022) proposed a new attention structure, which uses the characteristics of the feature pyramid to fuse the features of the adjacent lower layer to guide the upper layer to filter invalid features, so that the deep and shallow feature information is fully fused, and it improve the multi-scale target detection performance. Zhang and Slamu (2023) proposed a lightweight partial channel pooling attention mechanism, which selectively emphasizes interdependent channel mapping through the exchange of information between channels directly rather than through the convolution layer. Through a large number of experiments on object detection, it performs better on various types of basic models. In this way, the attention mechanism has been applied to plant disease recognition in complex scenes. Zhao et al. (2022) proposed a RIC-Net model which combines the improved convolution block attention module (CBAM). The recognition effect of corn, potato and tomato is good in the PlantVillage dataset. Wang et al. (2021) proposed an ADSNN-BO model based on MobileNet v3 and attention enhancement mechanism, and carried out cross-validation classification experiments based on an open rice disease dataset. There are four categories, which can achieve 94.65% test accuracy. Chen R. et al. (2022) proposed a model combining channel attention and channel pruning to reduce the parameters and complexity via the L1-norm channel weight and local compression ratio. The accuracy of model on the public dataset PlantVillage reaches 99.7% and achieves 97.7% on the local peanut leaf disease dataset.

The neural network method is effective for crop disease identification, and it has developed in recent years. Many studies have put forward new original networks, changed the network structure, reduced network computation, and enhanced the attention mechanism and fusion method. However, the existing neural network models can not accurately identify leaf diseases with highly similar features in fast and convenient effective way. Therefore, the research goal of this paper is to design a lightweight network model which can identify maize leaf diseases with various features heights under complex background.

Inspired by the above discussion, this study designed a convolution neural network model, namely, lightweight multi-scale feature network (LMFNet).The main innovations and contributions are summarized as follows:

1. A maize leaf disease dataset is established, including ten categories, namely, healthy leaf (healthy),northern leaf blight (nlb), gray leaf spot (gls), southern leaf blight (slb), corn rust(rust), curvularia leaf spot (cls),brown spot (bs), northern leaf spot (nls), autumn armyworm infection (fw) and zinc deficiency (zd). There are four categories, namely, cls,gls,nls and slb, with highly similar disease features.
2. The model uses partial convolution and varying expansion rates to build parallel multi-scale feature fusion module (PMFFM) achieving multi-scale feature extraction without requiring multiple convolution layers or pooling layers, and adds attention block (MAttion) to suppress complex background information to strengthen the disease features fusion at different scales.
3. The model outperforms some mainstream CNNs compared with it in all metrics, with the only 0.88M parameters. In addition, we also conducts experiments on the necessity of the PMFFM and MAttion module for the model using our dataset, verifing that it is essential. This article also conducts experiments to observe the disease features of maize leaves under complex background on the accuracy of the model.

The rest of this article is organized as follows. The “Materials and methods” section presents the dataset and methods adopted in this study. The “Experimental results and analysis” section presents the experiments for evaluating the performance of the model and analyzes the results of the experiments. Finally, the “Conclusion” section summarizes the main conclusions.

## 2 Materials and methods

### 2.1 Image acquisition

In this study, three ways were used to collect images of maize leaves, namely, open source crop disease dataset, public website and field photography. First of all, we looked up three open source crop disease datasets on the internet, namely CD&S (Ahmad et al., 2021), PlantDoc (Singh et al., 2020) and Corn-Disease(GitHub-FXD96/Corn-Diseases : Corn Diseases). These datasets provide the original images of maize leaf diseases with high resolution and complex background, and can reflect the real field situation. The CD&S dataset contains 1062 images of maize northern leaf blight, gray leaf spot and southern leaf spot, with a resolution of 3024 pixels × 3024 pixels. The PlantDoc dataset contains 300 images of corn rust with a resolution of 256 pixels × 256 pixels. The Corn-Disease dataset contains 323 images of maize leaves infected by armyworm and images of zinc deficiency, with a resolution of 300 pixels × 300 pixels. Secondly, we obtained the images of maize brown spot and Curvularia leaf spot from the public website (google,bing). These images come from different regions and environments and have good diversity and representativeness. A total of 111 images of corn brown spot were obtained with a resolution of 256 pixels × 256 pixels. A total of 117 images of corn Curvularia leaf spot were obtained with a resolution of 256 pixels × 256 pixels. Finally, we used an ordinary smartphone (Huawei smartphone VOG-AL00, manual focus) to collect 2000 images of maize leaf diseases in the natural environment of Hohhot, Inner Mongolia and Baoshan, Yunnan Province in June 21-31, 2023, and August 5-10, 2023. These images can reflect the effects of different climate and soil conditions on maize leaf diseases. After identification by agricultural technicians, we screened out clearly discernible images of maize leaf diseases, including healthy leaves, northern leaf blight, corn rust and southern leaf spot, which were 225,236,212 and 273, respectively. The resolution is 1080 pixels × 1920 pixels or 1080 pixels × 2340 pixels.

Through the above three ways, we collected a total of 3141 images of maize leaf diseases, covering the common types of maize leaf diseases, providing rich data resources for subsequent identification and classification of maize leaf diseases. As the same disease is divided into general and serious symptoms, the inter-class variance in the dataset is small. On the other hand, in our dataset, there are leaf images with highly similar disease features, but not belong to the same disease which had a true label identification by agricultural technicians. So it is easy due to label different from the actual classification, which affects the evaluation of the CNN model. Therefore, it is challenging for a CNN model to identify the disease accurately.

### 2.2 Image preprocessing

**Figure 1:**
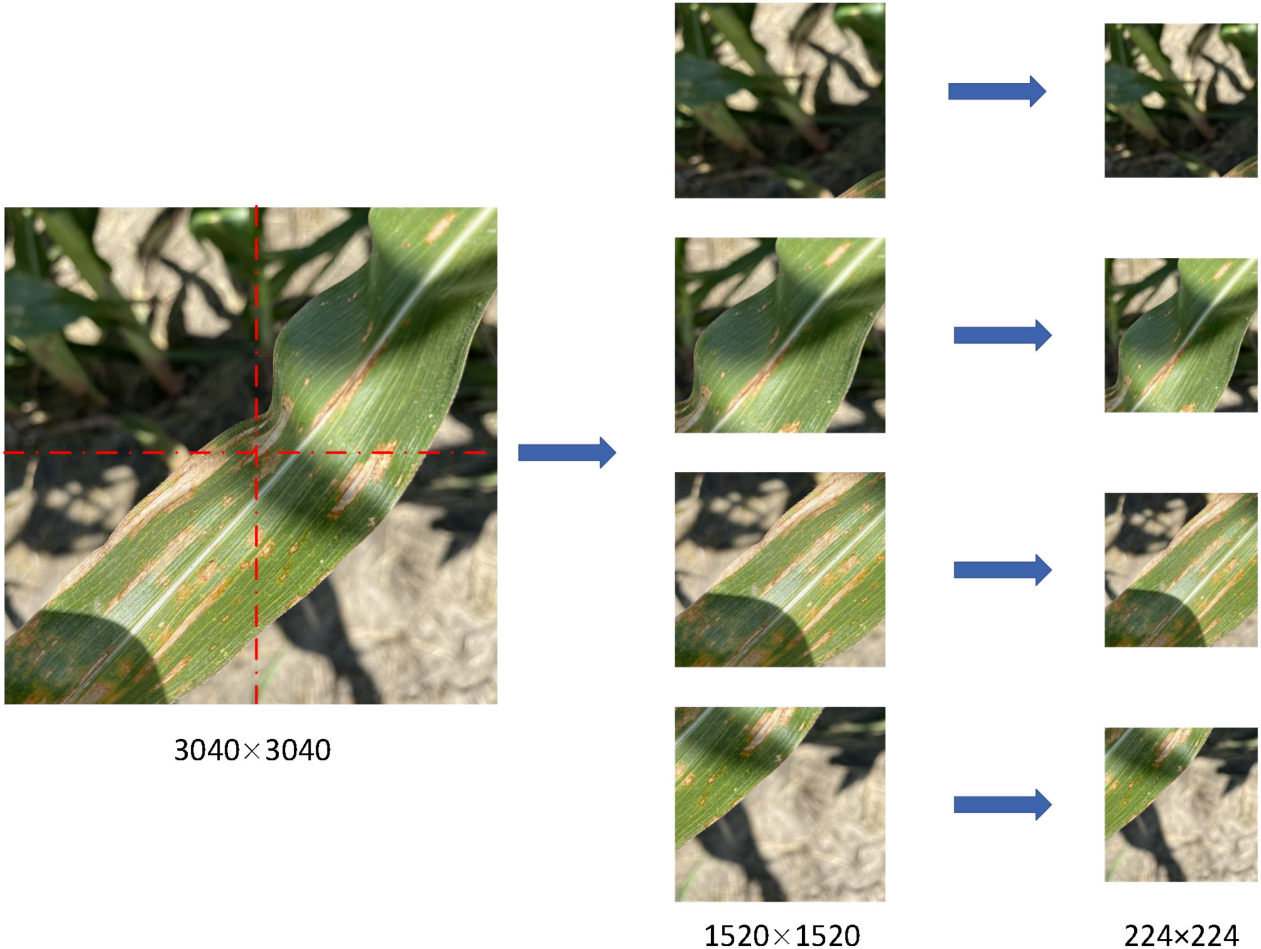
Original images are cropped and resized.

To enhance the utilization and diversity of maize leaf disease images, we cut the images with higher resolution and get more sub-images. The specific cutting methods are as follows: firstly, we selected the images with maize northern leaf blight, gray leaf spot, northern leaf spot, corn rust and southern leaf spot, as well as healthy leaf images. these images come from CD&S datasets and field data, with a resolution of 3024 pixels × 3024 pixels or 1080 pixels × 1920 pixels or 1080 pixels × 2340 pixels. Then, according to the length of the long and short edges of the image, we determine that the cutting length is half of the longer and shorter edges, that is, 1512 pixels, 540 pixels or 1170 pixels. Then, starting from the center point of the image, we cut to both sides along the long edge and the short edge, respectively, and get four sub-images of the same size. the resolution of each sub-image is 1512 pixels × 1512 pixels or 1170 pixels × 1170 pixels. Finally, we save the cut sub-image as a new file for subsequent use and analysis. The schematic diagram of the cutting process is shown in **Figure 1**. Because part of the image is cut, the image contains a lot of background information, and there are no effective disease leaves, so it is necessary to screen the image and eliminate the interference.

The number of maize disease samples was limited, and the number of samples of different categories was not evenly distributed. To reduce overfitting during model training and enhance the generalization ability of the model, the dataset had to be expanded. The specific processing methods are as follows: firstly, we enhanced the images of maize brown spot, northern leaf spot, zinc deficiency and autumn armyworm infection. These images come from public websites and Corn-Disease datasets, and there are only 111,117,179 and 144images with a resolution of 256 pixel × 256pixel or 300pixel × 300pixel. We apply five methods of data enhancement, namely random rotation, random offset, crosscutting transformation, random scaling and random flipping, in order to increase the change and difficulty of the image and improve the generalization ability of the model. Then, we adjust the resolution of all the maize leaf disease images uniformly, and adjust the size of the image to 224 pixels × 224 pixels to meet the input requirements of the image classification network. Then, according to the proportion of 8:2, we divide the dataset into training set and verification set. The training set is used to train the image classification network, and the verification set is used to evaluate the performance and effectiveness of the network. Finally, we counted the types of maize leaf diseases and the number of images, as well as the number of images in training set and verification set. The results are shown in **Table 1**.

**Table 1:** Number of specific categories and distribution of training and test datasets.

Disease type	original	expend	Train set	Val set
Healthy	225	900	720	180
Gray leaf spot	248	962	776	186
Brown spot	111	666	531	135
Northern leaf bright	497	1205	960	245
Northern leaf spot	553	2112	1700	412
Curvularia leaf spot	142	852	680	172
Southern leaf blight	273	1092	876	216
rust	512	1148	918	230
zinc deficiency	179	1074	860	214
armyworm	144	864	690	174

### 2.3 LFMNet model

#### 2.3.1 The structure of model

**Figure 2:**
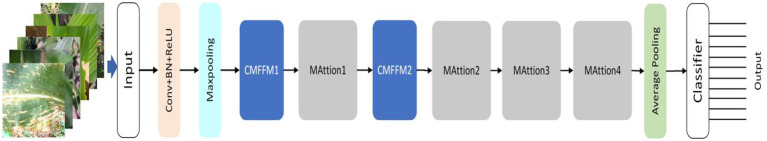
LFMNet model.

LFMNet is a deep learning network that identifies maize leaf diseases in the natural environments. It has two main modules: the parallel multi-scale feature fusion module(PMFFM) and the attention mechanism module(MAttion). The PMFFM uses expansion convolution to extract features of maize leaf diseases at different scales, which enhance the receptive field and expression ability of the network. The MAttion uses the attention mechanism to locate the position and extent of maize leaf diseases accurately. The structure of the LFMNet network is shown in **Figure 2**. The input of LFMNet network is a 3-channel image of maize leaf disease, with a size of 224 pixels × 224 pixels. The output of the network is a classification result of 10 categories, indicating the type of maize leaf disease or healthy leaf in the image. The network works as follows: first, it uses a 7 × 7 convolution layer and a 3 × 3 maximum pool layer to down-sample the input image and obtain a 24-channel feature map with a size of 56 pixels by 56 pixels. Then, it uses the PMFFM to extract features from the feature map. The PMFFM has three partial convolution layers with different expansion rates (1, 2, and 3) to extract features at different scales. The input and output feature map dimensions of the PMFFM are the same. The MAttion has a 1 × 1 convolution layer, a 3 × 3 maximum pooling layer and two sub-modules that process the feature map in parallel: the PPA block and the MSA block. The PPA block is a partial channel attention block that splits the input feature map into several parts, pooling some features, obtaining local features and reorganizing them. Then, it concatenates the local extracted features with the rest to generate a new feature map that enhances the ability of the network to learn image features. The MSA is a multi-layer attention block that has three branches. The first and second branches focus on global information, and the third branch focuses on local information. The branches use different pooling kernels to explore different clues of feature information and compute the weight of each channel on each branch. Then, they combine the weights of the three branches to obtain the global feature weight and focus on the disease feature region. The MAttion1 module outputs a 48-channel feature map with a size of 28 pixels by 28 pixels. The MAttion2 module outputs a 96-channel feature map with a size of 14 pixels by 14 pixels. The MAttion3 module outputs a 192-channel feature map with a size of 7 pixels by 7 pixels. The MAttion4 module outputs a 256-channel feature map with a size of 3 pixels by 3 pixels. Finally, the network uses a 1 × 1 average pooling layer to pool the feature map globally and obtain a 256-dimensional feature vector. Then, it uses a 256 × 10 fully connected layer to classify the feature vector and produce a 10-dimensional classification vector. The architecture definition of the LFMNet network as shown in **Table 2**. PConv stands for partial convolution.

**Table 2:** LFMNet structure.

Layer name	Output tensor	Configuration	Parameters
Input	3×224×224	Augmented images	0
Conv_1	24×112×112	Conv(k=7, s=2),BN,ReLU	3576
MaxPool	24×56×56	k=3,s=2	0
PMFFM1	24×56×56	[PConv(k=3),BN,ReLU]×3,dilation=1,2,3	1116
MAttion1	48×28×28	Conv(k=1, s=1),BN,ReLU MaxPool k=3,s=2,PPA, MSA[AveragePool(k=3),MaxPool(k=5), Conv(k=1,s=1),PConv(k=3),ReLU]	22389
PMFFM2	48×28×28	[PConv(k=3),BN,ReLU]×3,dilation=1,2,3	4176
MAttion2	96×14×14	Conv(k=1, s=1),BN,ReLU MaxPool k=3,s=2,PPA, MSA[AveragePool(k=3),MaxPool(k=5), Conv(k=1,s=1),PConv(k=3),ReLU]	98112
MAttion3	192×7×7	Conv(k=1, s=1),BN,ReLU MaxPool k=3,s=2,PPA, MSA[AveragePool(k=3),MaxPool(k=5), Conv(k=1,s=1),PConv(k=3),ReLU]	352704
MAttion4	256×3×3	Conv(k=1, s=1),BN,ReLU MaxPool k=3,s=2,PPA, MSA[AveragePool(k=3),MaxPool(k=5), Conv(k=1,s=1),PConv(k=3),ReLU]	442880
AveragePool	256	k=1	0
Classifier	10		2570
Total			924953

#### 2.3.2 PMFFM block

**Figure 3:**
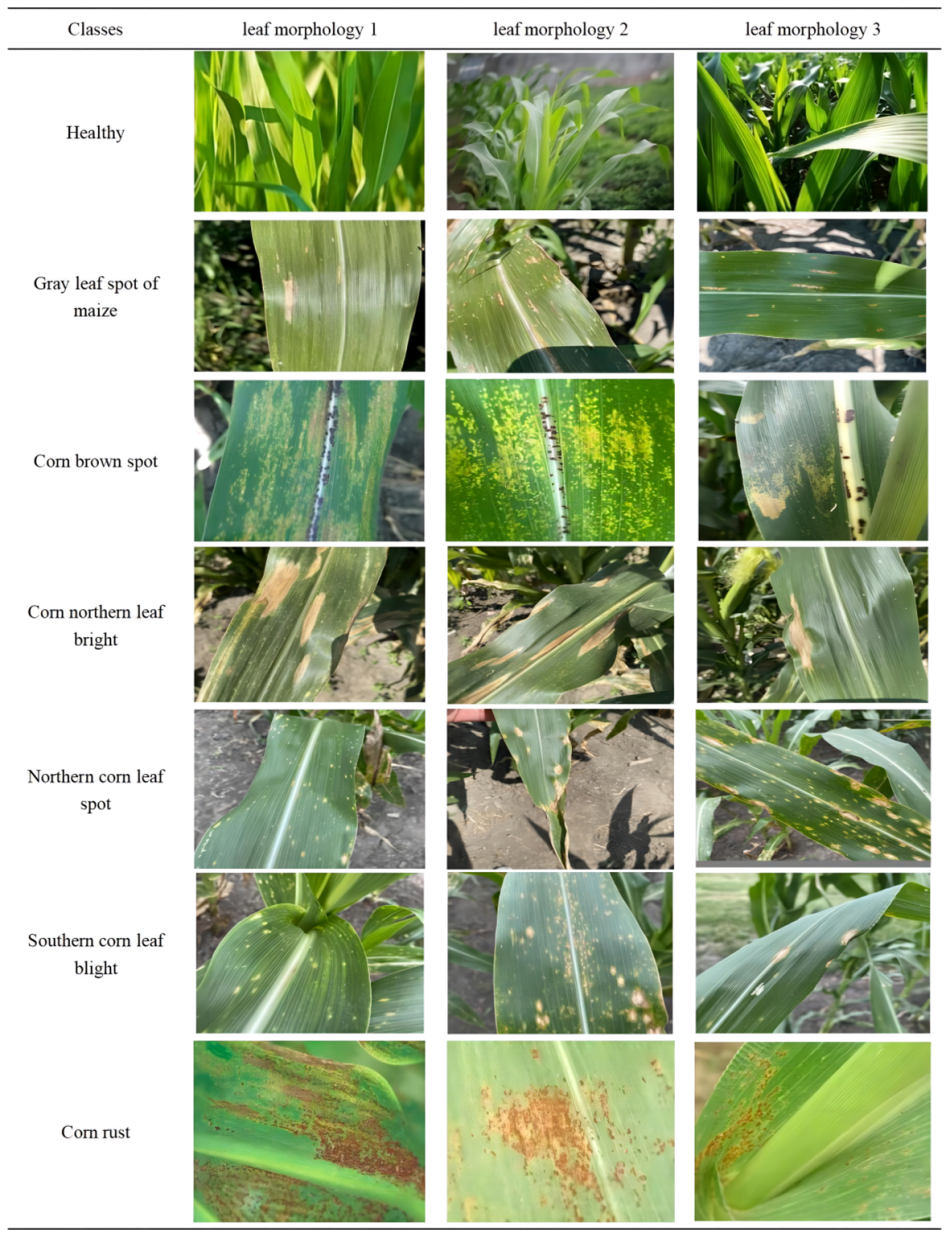
Morphology of maize leaf diseases.

Leaf diseases have complex symptoms and morphological features in different growth stages and scales. Sometimes, different diseases have similar features at the same scale. For example, northern leaf blight can manifest as single spots or clustered spots, as shown in **Figure 3**.

**Figure 4:**
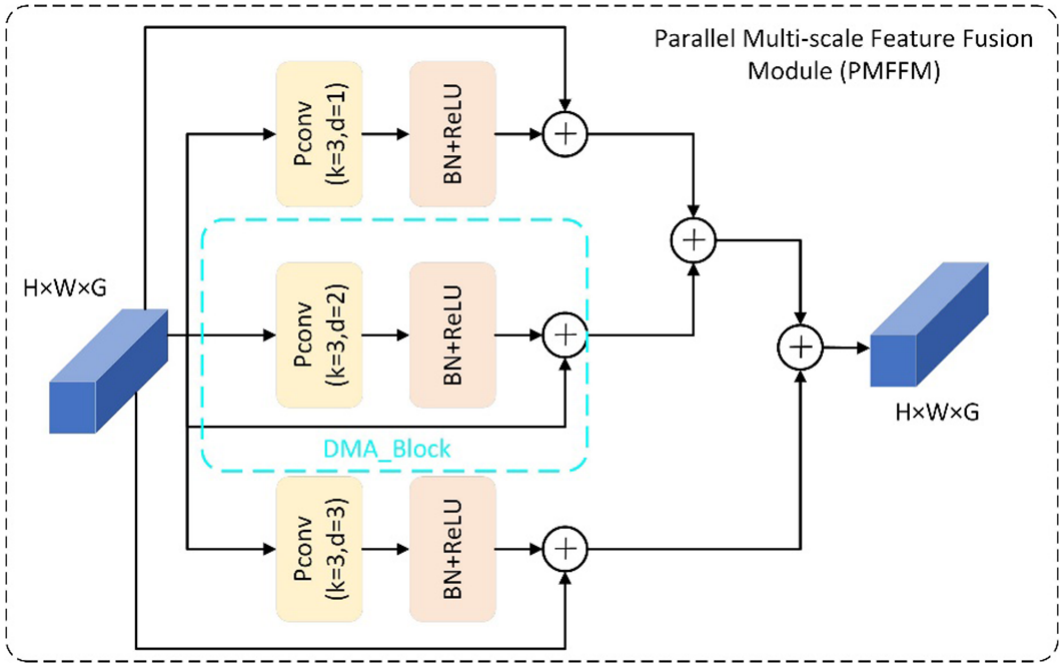
PMFFM block.

To identify this disease, we need to look at the large-scale and coarse-grained features of the leaf. On the other hand, northern leaf spot and southern leaf blight are both characterized by round and scattered spots. To distinguish them, we need to examine the small-scale and fine-grained features of the leaf. Therefore, the multi-scale information of leaf disease features in the image is crucial for accurately identifying the types of maize leaf diseases. The PMFFM module is a type of module that extracts multi-scale features of maize leaf diseases. It is inspired by the GMDC module (Zeng T. et al., 2022). which uses group convolution and multi-scale feature extraction to increase the receptive field and expression ability of the network. The PMFFM module improves on the GMDC module by using partial convolution and varying expansion rates to achieve multi-scale feature extraction, without requiring multiple convolution layers or pooling layers. This reduces the model’s parameters and computation. **Figure 4** shows the structure of the PMFFM module. The PMFFM module consists of three parallel DMA_Block modules. Each DMA_Block module uses a 3×3 partial convolution layer with a different expansion rate (1, 2, or 3) to extract features at different scales from the input. The feature map is then normalized and activated by a batch normalization layer and a ReLU activation function to improve the stability and nonlinearity of the features. Finally, the input and output features are added together to form a new feature map using skip connections. The PMFFM module fuses the feature maps of the three DMA_Block outputs to obtain the global and detailed information of different symptoms and morphological characteristics of maize leaf disease. The input and output feature map dimensions of the PMFFM module are the same. The expansion rate is chosen based on the experiment of expansion convolution (Zeng et al., 2022b). When the expansion rate is 1, the pixel information of the original feature map is preserved at the top layer, thus avoiding the loss of information due to the excessive expansion rate in the middle layer.

#### 2.3.3 MAttion block

The MAttion block is an attention module that locates maize leaf diseases. It has a point-wise convolution layer that down-samples the input feature maps and two attention modules that process them in parallel.

**Figure 5:**
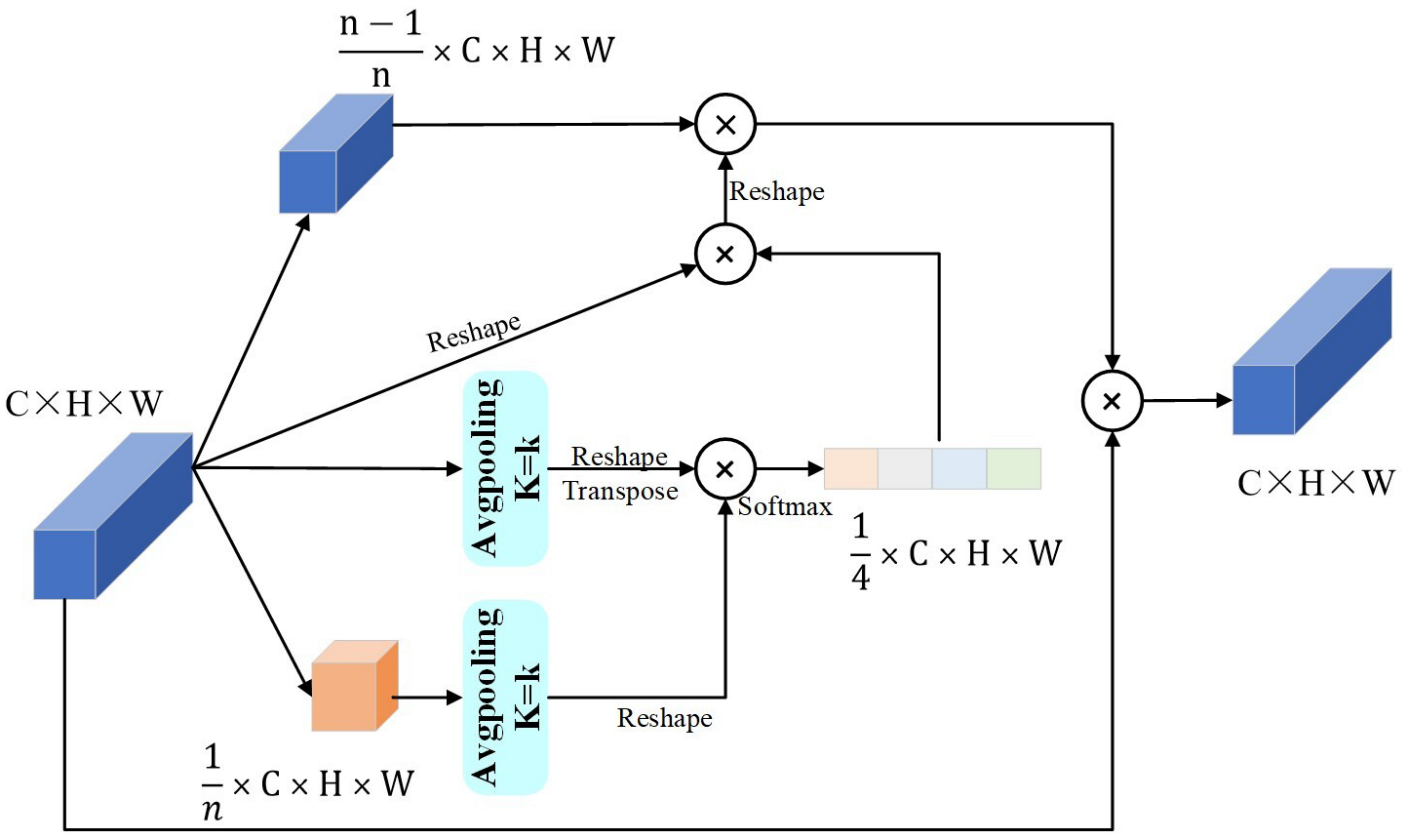
PPA structure.

We modify the PPA structure (Zhang and Slamu, 2023) as shown in **Figure 5**. The main improvements are: first, it did not limit the slice size of the input feature map; second, it adapted the output size of the pooling operation for the smaller part of the slice and the input feature map, based on the input feature map size, because the model uses the PPA many times and the input feature map size changes each time. Third, it did not use the convolution operation of the original model to synthesize the new feature map, but it useed the reshape operation, so the parameters reduced while extracting the same features.

The pooling kernel size depends on the number of MAttion blocks. We make a list of the number of MAttion blocks. Let *N* be the number of modules, and *i* ϵ [MAttion_n_ | n ϵ 1,2,3,4,…n], *i* be the module number, starting from 1. In Equation 1, k is the pooling kernel size. The formula shows that the more MAttion blocks there are, the larger the pooling kernel size for each block. Equation 2 *X_c_* (*m,n*) shows the size of each pixel of the input feature map and *Z_c_* (*i,j*) shows the size of the feature map pixels after the pooling operation with the kernel size k.


(1) $$k=3+2\times i,\;i\in [MAttion_{n}\;|\;\text{n}\in 1,2,3,4,\ldots ,\text{n}]$$

(2) $$Z_{C}\left(i,j\right)=\frac{K^{2}}{HW}\sum_{m=\frac{wi}{k}}^{\frac{w\left(i+1\right)}{k}}\sum_{n=\frac{hi}{k}}^{\frac{h\left(i+1\right)}{k}}X_{C}\left(m,n\right),\;$$

**Figure 6:**
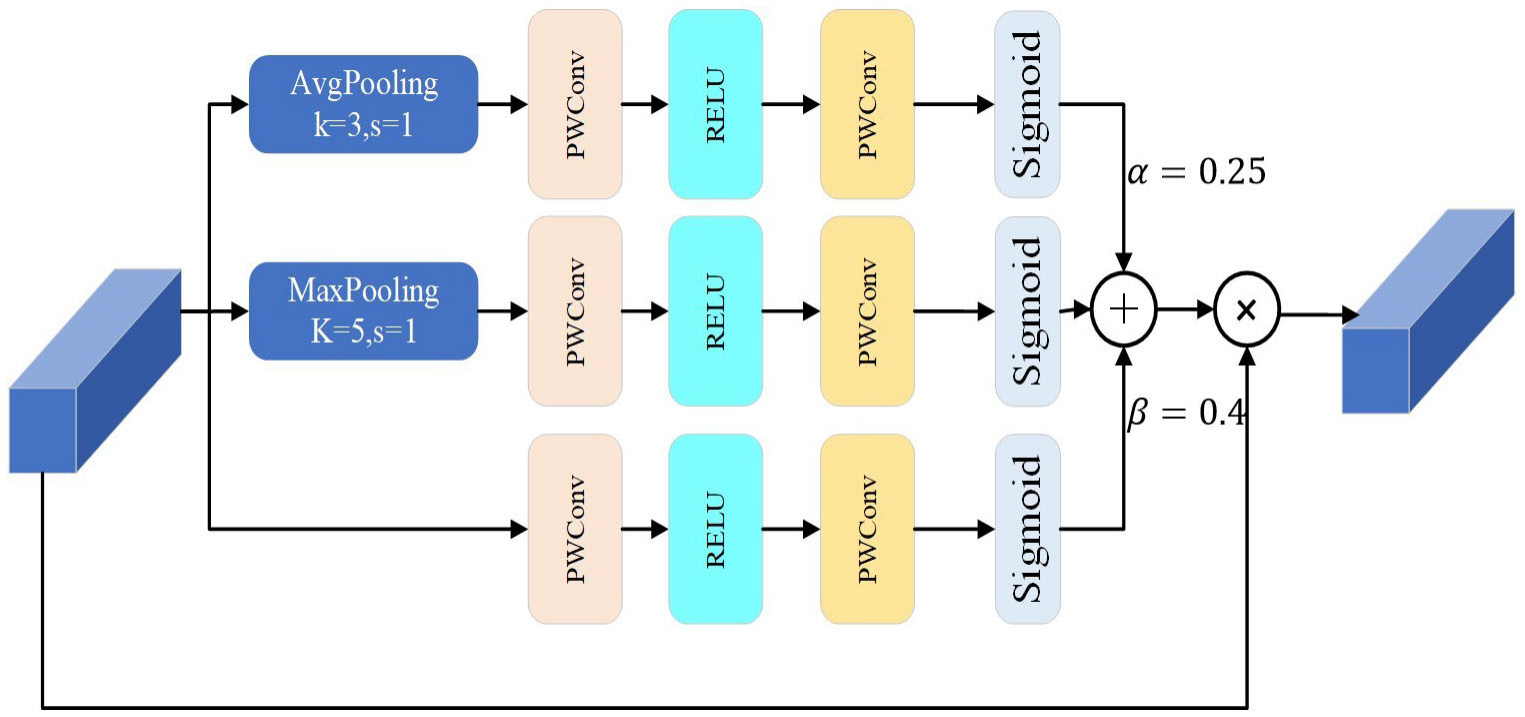
MSA block.

The MSA structure, as shown in **Figure 6**, is an improvement on MWAB (Gao and Zhou, 2023). It had three pooling operation branches with different kernel sizes for the average and maximum pooling layers. The original model uses a kernel size of 3 for the average pooling layer and 5 for the maximum pooling layer. To keep the input feature map size unchanged, the MSA structure uses a kernel size of 3 with padding 2 for the maximum pooling layer and replaces the kernel size of 5. The experiments on the model show that the kernel sizes of 3 and 5 are the most effective ones. These changes help extract important features at different scales and transform them into feature weights using sigmoid functions. The feature weights at different scales are combined to form a complete feature weight matrix, which is multiplied by the input feature map to produce the output feature map of the key features.

From the theoretical analysis, it is assumed that the input of MSA is that X = [x_1_,x_2_,…,x_c_,…,x_c_] ϵ ℝ^H×W×C^,the weights of the three branches are expressed by the **Equation 3**:


(3) $$\{\begin{array}{c} W_{1}=\sigma \left(F_{pwc1\times 1}\left(F_{pc3\times 3}\left(F_{GAP3\times 3}\left(x\right)\right)\right)\right)\\ W_{2}=\sigma \left(F_{pwc1\times 1}\left(F_{pc3\times 3}\left(F_{GMP5\times 5}\left(x\right)\right)\right)\right)\\ W_{3}=\sigma \left(F_{pwc1\times 1}\left(F_{pc3\times 3}\left(x\right)\right)\right)\;\;\;\;\; \end{array}$$

where σ() is the sigmoid function, and *F_pc_* () is the 3 × 3 part convolutional function, *F_pwc_* () is a 1 × 1 pointwise convolution function. *F_GAP_*_3×3_ is the global average pooling function, and *F_GMP_*_5×5_is the global max pooling function.

The output *F_MSA_* of the MSA can be described by the **Equation 4**:


(4) $$F_{MSA}=\left[\alpha W_{1}+\left(1-\alpha -\beta \right)W_{2}+\beta W_{3}\right]⊙x$$

where ⊙ is the element-wise multiplication operation.α, β are proportional parameters.

## 3 Experimental results and discussion

### 3.1 Experimental configuration and analysis

The experimental hardware in this study used Ubuntu operating system and Intel ^®^Xeon ^®^Platinum 8255C processor (2.50GHz). The model training and testing were accelerated by GPU, and the GPU model was NVIDIA RTX 3090 24GB. The software environment used Python 3.8, Cuda 11.3 and Pytorch 1.11.0 frameworks. The related experiments were performed using the experimental data. The experiment consisted of three parts, namely, the comparison of different network models, the comparison of experiments on different datasets, and the ablation experiment.

When training the maize leaf disease identification model, we used SGD (Stochastic Gradient Descent,SGD) to optimize the network model. SGD algorithm was relatively stable when adjusting training parameters, and had small memory requirements; therefore, it was suitable for most non-convex optimization problems. The learning rate was set to 0.001, the momentum parameter was set to a fixed value of 0.6, the number of iterations was epochs = 800, and the number of images entered in each batch was batch size = 32. All models use the weight initialization strategy in (He et al., 2015) work.

### 3.2 Evaluation indexes

To show the performance of the network in this study, refer to the model evaluation indicators in (Xie et al., 2018) work, we select accuracy(Acc), precision(P), recall(R), F1-score(F1), parameters and floating-point of operations(FLOPs) to evaluate the performance of the network model in the identification of maize leaf disease. These measurement indicators can be calculated by the following **Equations 5**–**8**:


(5) $$Acc\;=\;\frac{TP+TN}{TP+FP+TN+FN}$$

(6) $$P=\;\frac{TP}{TP+FP}$$

(7) $$R=\;\frac{TP}{TP+FN}$$

(8) $$F1=\;2\times \frac{P\cdot R}{P+R}$$

where TP, TN, FP, and FN are the number of true positive samples, true negative samples, false-positive samples, and false-negative samples, respectively. P estimates how many of the predicted positive samples is positive. The R is the assessment of how many of all positive samples can be correctly predicted as positive. F1 is the synthesis of precision and recall. Acc measures global sample prediction. Parameters, and FLOPs are commonly used to measure model complexity.

### 3.3 Comparative experiment on different network models

**Figure 7:**
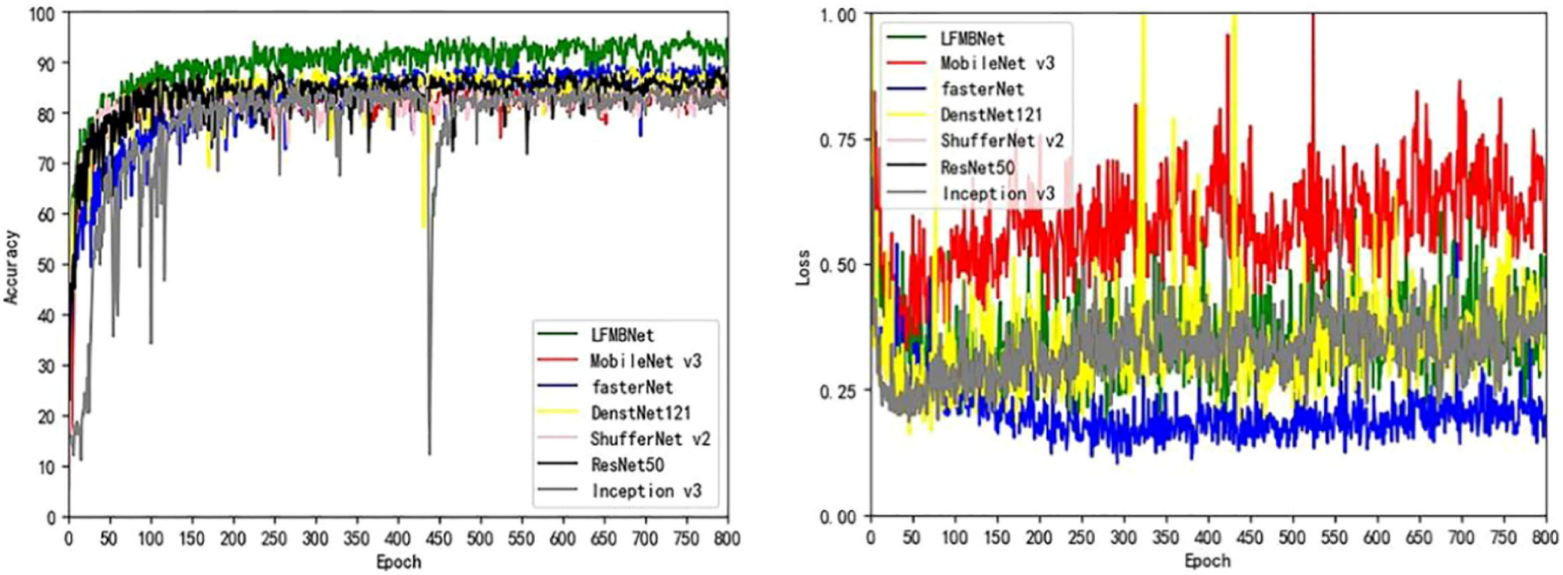
Comparison of experimental results of models.

We compared LFMNet with the common maize leaf disease identification model on the maize leaf disease dataset that we proposed in this paper. As shown in the **Table 3**, the proposed network model was tens or even hundreds of times higher than the heavyweight network (ResNet 50, FasterNet) in terms of parameters (Params) and FLOPs. Moreover, it had the highest average Acc,Precision, recall and F1 scores (about 7 per- cent higher than ResNet 50). Comparing with several commonly used lightweight networks (Densenet-121, MobileNet V3-large, ShuffleNet V2), the proposed model had the fewest parameters, and improved the average Acc, Precision, recall rate and F1 score. As shown in **Figure 7**, the LFMNet had better than other models at overall performance. However, there was a abnormal situation that the LFMNet had higher accuracy than the fasterNet model but higher loss than the fasterNet model. This may be due to not correct classification when identifying diseases with highly similar features, the fasterNet distinguish different categories are divided into the same category resulting the average accuracy low, but the overall loss is reduced.

**Table 3:** Comparison of recognition accuracy of different models.

Models	Precision	Recall	F1_score	Accuracy	Parameters(M)	FLOPs
MobileNetv3	84.73	84.86	84.53	84.86	1.67	64.85M
FasterNet	90.21	90.08	90.06	90.08	31.18	4.5G
DenseNet121	90.15	90.24	90.11	90.24	6.96	2.9G
ShufferNet v2	86.05	86.13	85.87	86.13	1.26	151.37M
ResNet50	89.39	89.61	89.24	89.61	23.53	4.13G
Inception v3	86.58	86.92	86.62	86.92	23.46	3.09G
LFMNet	94.26	94.12	94.09	94.12	0.88	45.78M

**Figure 8:**
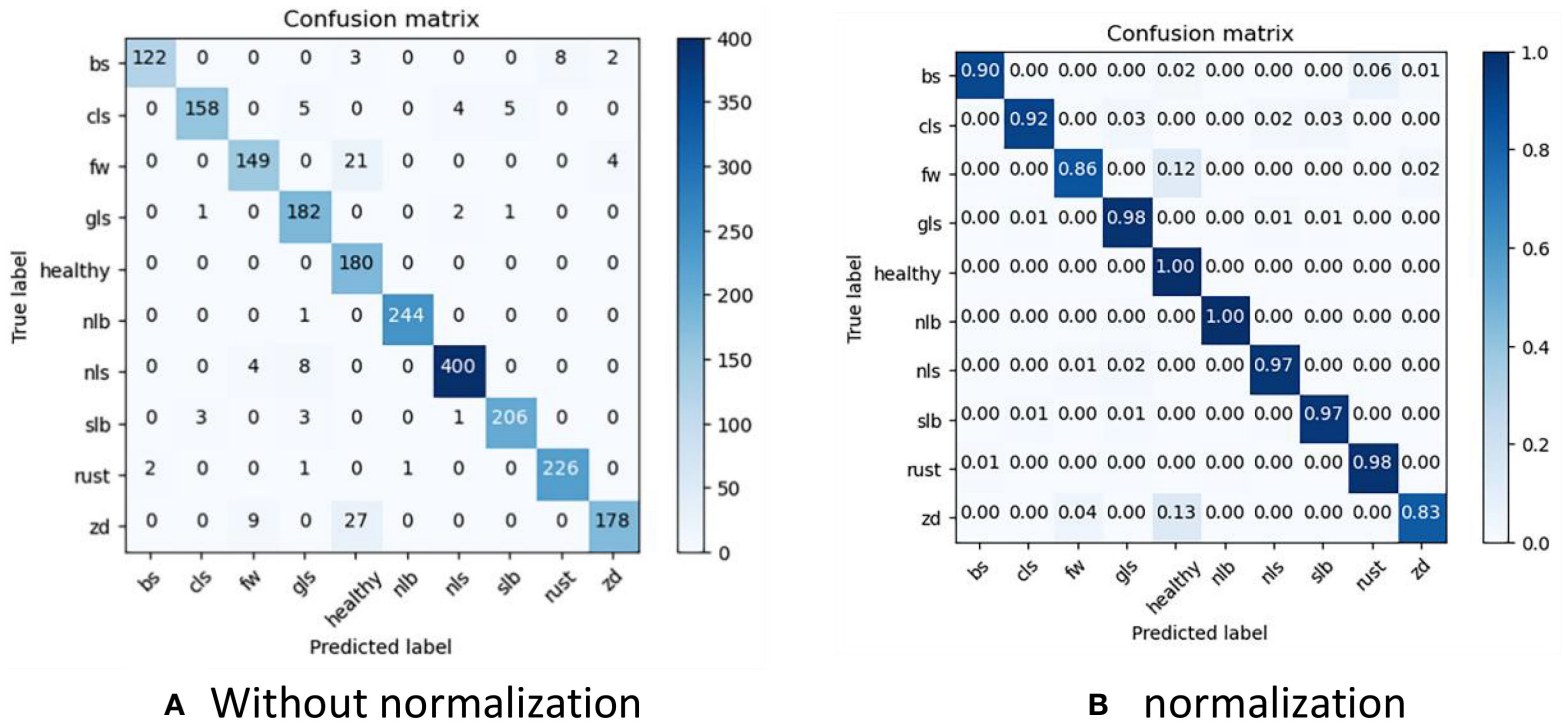
Obfuscation matrix.

To clearly present the results of LFMNet recognition accuracy, we drew a confusion matrix based on the dataset we built, as shown in **Figure 8**. Bs, cls, fw, gls, nlb, nls, slb, rust and zd represent nine common maize leaf diseases, and they are acronyms for the nine maize diseases listed in **Table 1**. Considering the simple background of the Healthy image, it was easy to identify, with an accuracy of 100%. The background of nlb was complex, but the disease spot was single and wide, which wass favorable for the recognition of our model, with an accuracy of 99.9%, and only one image was recognized as gls. On the other hand, the area of rust disease was large and scattered, but the features were obvious, which was also beneficial for the identification of our model, with an accuracy of 98%. For slb, nls and gls, the background of the original image was complex and disease-intensive and diverse, the features of disease spots were highly similar, the recognition process had errors, and the overall recognition accuracy is more than 97%. We believe that the error was mainly caused by the concentration of disease spots and mixed categories, which made these categories more difficult to identify than other categories. Zd image recognition, because the disease spot feature was not obvious and was not different from the background, was easily disturbed by the complex background, resulting in serious confusion, with only 83% accuracy. In addition, the indexes of this model for identifying various maize leaf diseases are listed in **Table 4**. “support” represents the number of images.

**Table 4:** Identification of maize leaf disease index by LFMNet.

	Precision	Recall	F1_score	Support
bs	0.9136	0.9023	0.8985	135
cls	0.9899	0.9245	0.9561	172
fw	0.9029	0.8575	0.8796	174
gls	0.9217	0.9770	0.9485	186
healthy	0.9914	1.0000	0.9955	180
nlb	0.9593	0.9992	0.9583	245
nls	0.9700	0.9700	0.9700	412
slb	0.9896	0.9700	0.9797	216
rust	0.8850	0.9800	0.9245	230
zd	0.8711	0.8258	0.8478	214
accuracy			0.9412	2164
macro avg	0.9422	0.9323	0.9359	2164
weighted avg	0.9426	0.9412	0.9409	2164

### 3.4 Ablation experiments

We conducted ablation experiments on the same dataset to compare various PMFFM and different combinations of attention mechanism optimizations. First, without using attention mechanism optimization methods, we employed two PMFFM (Parallel Multi-Feature Fusion Module) blocks as the base model. Based on this base model, we designed four combinations: LMFNet-V0 (PMFFM=2), LMFNet-V1 (PMFFM=3), LMFNet-V2 (PMFFM=4), and LMFNet-V3 (PMFFM=5) to evaluate our constructed dataset. The experimental results revealed that as the number of cascaded PMFFM blocks increased, the accuracy exhibited a downward trend. The reason behind this lies in the structure of the PMFFM blocks. While multi-scale feature extraction allows us to capture as many disease-related features from images as possible, an excessive number of cascaded PMFFM blocks limits the fine-grained extraction of disease features. This can lead to parameter redundancy, wastage of computational resources, and a decrease in accuracy due to overfitting. Generally, an appropriate number of PMFFM blocks can effectively enhance recognition accuracy without significantly increasing computational complexity. Next, under the same PMFFM module combination, we compared the MAttion attention module with the PPA (Positional Pyramid Attention) and MSA (Multi-Scale Attention) modules. Experimental results across all models showed that the MAttion module in LMFNet-V0 achieved the highest recognition accuracy. It outperformed the non-fused MAttion module in LMFNet-V0 by 2.58% and surpassed the hybrid PPA and MSA modules in LMFNet-V0 by 1.84% and 3.14%, respectively. This indicates that the MAttion attention module, which combines PPA and MSA, improves the recognition of corn leaf diseases in complex backgrounds more effectively than individual PPA or MSA modules. Overall, our proposed feature extraction combination in this study effectively identifies corn leaf disease features. As shown in **Table 5**, the model constructed using two PMFFM modules and the MAttion attention fusion mechanism achieved the highest precision, recall, and F1 score.

**Table 5:** Network model structure and comparative experiment on different optimization methods.

Model	PMFFM	PPA	MSA	MAttion	Precision	Recall	F1_score	Accuracy
LFMNet-v0	2	√			92.42	92.66	91.94	92.66
			√		91.12	91.56	92.01	91.56
				√	94.26	94.12	94.09	94.12
					91.68	91.14	91.17	91.14
LFMNet-v1	3	√			91.23	92.44	92.05	92.44
			√		90.32	89.90	90.21	89.90
				√	93.16	93.65	93.99	93.65
					88.52	87.82	87.74	87.82
LFMNet-v2	4	√			92.12	92.99	92.34	92.99
			√		89.87	90.23	90.19	90.23
				√	91.11	91.52	90.87	91.52
					89.43	88.61	88.62	88.61
LFMNet-v3	5	√			86.14	86.39	85.93	86.39
			√		85.45	85.98	84.78	85.98
				√	87.67	88.45	88.25	88.45
					87.20	85.60	85.41	85.60

**Figure 9:**
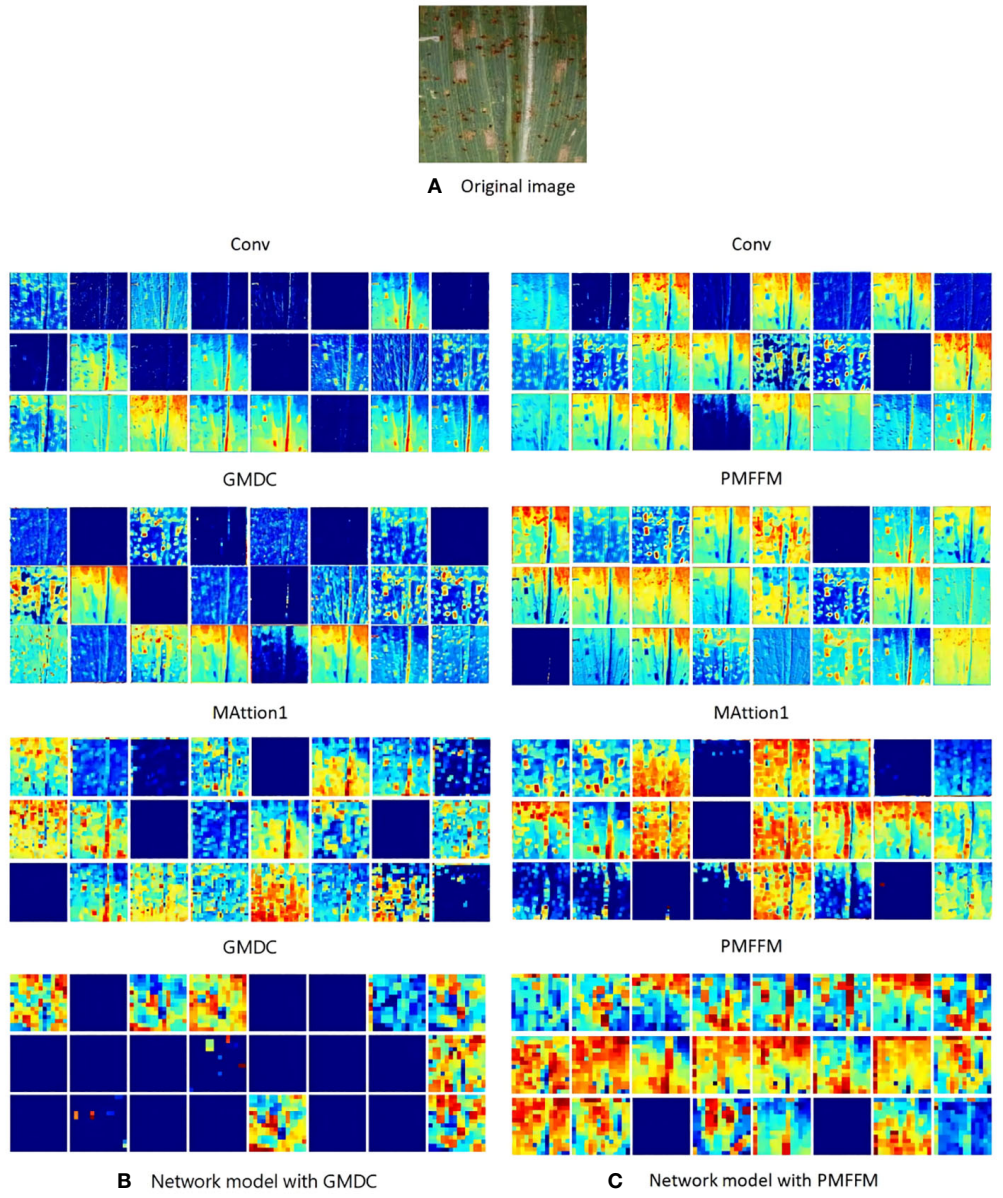
Visualization results of the output feature maps of the convolution layer.

The PMFFM module is an enhanced and optimized version of the GMDC module. To verify the impact of the PMFFM module on the network model, we visualized the output feature maps of the models using the GMDC module and the PMFFM module respectively. As shown in **Figure 9**, the network model extracts the texture, color, and edge of the maize leaf disease in the Conv layer.As the network depth increases, the extracted features become more abstract. We observed that the LFMNet with the PMFFM module has richer abstract information than the LFMNet withthe GMDC module. This is because the PMFFM employs partial convolution and diverse dilation rates to capture more scale features, while preserving a large amount of detail features, thereby enhancing the model’s ability to recognize maize leaf disease.

**Figure 10:**
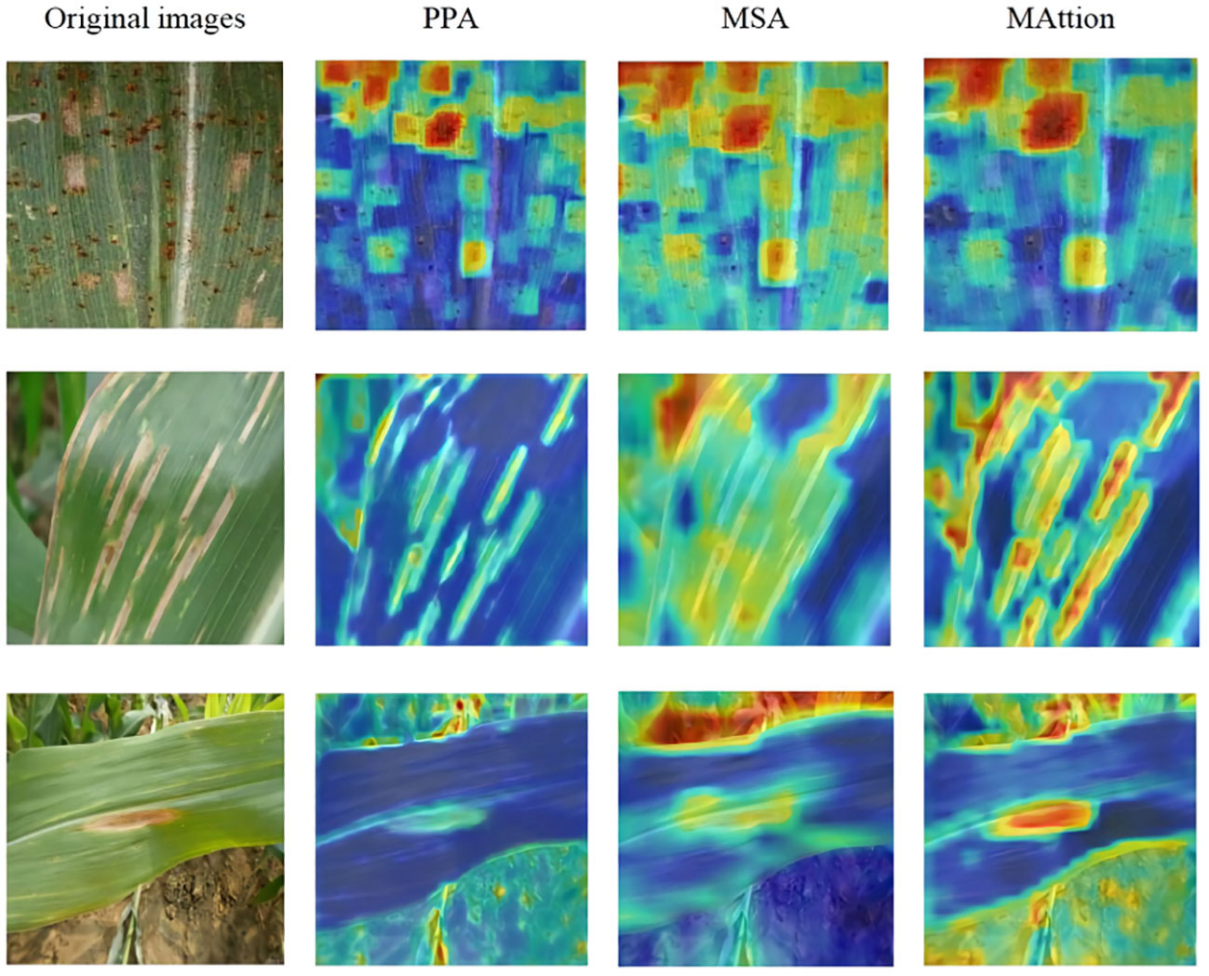
ScoreCAM visualization results.

To demonstrate that the MAttion proposed in this study can improve the effectiveness of identifying maize leaf disease under complex backgrounds, we visualized the three fusion methods using ScoreCAM (Wang et al., 2019), as shown in **Figure 10**. The first picture on the upper left shows maize leaf rust from the Plant Village dataset; the background of the dataset is relatively simple, but the disease distribution is dense. The disease spots in the picture are mostly located at the edge,scattered, and there is also the interference from gray leaf spots. Through identifying the disease spot with PPA and MSA, MAttion can effectively grasp the details of rust and identify the dense rust spot. The second picture shows maize leaves with southern leaf blight disease, taken with a conventional smartphone under a complex background in a field. The image background interference is strong, the disease spots are elongated, and there are a large number of disease spots on the edge of the leaf. PPA focuses on the disease spot area more effectively than MSA, while MAttion can accurately identify maize leaf diseases in the presence of background interference. The third picture shows northern leaf blight, which has a more complex background. As the color of the disease spot is similar to that of the land, it causes a lot of interference. While identifying the spots, MAttion can see that part of the background is also represented. On the whole, it can be seen that the disease spots recognition effect of MAttion enhanced by PPA and MSA is better and more accurate.

### 3.5 Comparison of experiments performed on different datasets

To demonstrate the high accuracy of our proposed method on different datasets, we compare LFMNet with the lightweight model by Bhuiyan et al. (2023) on the AI Challenger 2018 dataset, which contains seven types of grape leaves. The BananaSqueezeNet is a fast and lightweight CNN architecture that was optimized with Bayesian Optimization. It can also recognize plant leaves with similar disease features as LFMNet, which is why we chose it as a baseline. Furthermore, we evaluated the performance of the two models on our own dataset which we constructed for this study. The average accuracy of identification is shown in **Table 6**. LFMNet outperforms BananaSqueezeNet, indicating that our method has better identification performance.

**Table 6:** Comparative evaluation of experiments performed on different datasets.

Datesets	Model	Precision	Recall	F1_score	Accuracy
AI Challenger 2018 datasets	BananaSqueezeNet	94.13	94.74	95.10	94.74
	LFMNet	96.42	97.02	96.04	97.02
Our proposed datasets	BananaSqueezeNet	90.01	90.10	89.96	90.46
	LFMNet	94.26	94.12	94.09	94.12

## 4 Conclusion

In this article, LFMNet was proposed for similar diseases features of maize leaves under complex background recognition. In our method, a PMFFM module is responsible for identifying maize leaf diseases at different scales using different expansion rates. In the next phase, the MAttion module is used to fuse attention features to enhance recognition effect by combining the PMFFM and the MAttion to build the fine-grained LFMNet model. To verify the effectiveness and robustness of the model, experiments were conducted on the constructed maize leaf disease dataset and AI Challenger 2018 datasets and compared with the lightweight and classical CNN models, such as ResNet50,MobileNetV3, FasterNet, DenseNet121 and ShuffleNetV2. The recognition accuracy of the model is 94.12 and 97.02%, which is the highest.

In future work, we plan to deploy LFMNet on mobile devices such as field robots and unmanned aerial vehicle to establish an automated disease detection platform. In addition, to extend LFMNet’s applicability on disease identification of other plants, we will consider expanding its disease identification types through transfer learning.

## Data availability statement

The raw data supporting the conclusions of this article will be made available by the authors, without undue reservation.

## Author contributions

JH: Methodology, Validation, Visualization, Writing – original draft. XJ: Writing – review & editing. JG: Conceptualization, Data curation, Writing – review & editing. XY: Data curation, Investigation, Writing – review & editing.
